# Construct validity and reliability of the generalised anxiety disorder-7 scale in a sample of tuberculosis patients in the Free State Province, South Africa

**DOI:** 10.4102/sajid.v36i1.298

**Published:** 2021-08-19

**Authors:** Gladys Kigozi

**Affiliations:** 1 Centre for Health Systems Research & Development, Faculty of the Humanities, University of the Free State, Bloemfontein, South Africa

**Keywords:** tuberculosis, GAD-7, primary healthcare, anxiety, construct validity, confirmatory factor analysis

## Abstract

**Background:**

Generalised anxiety disorder (GAD) frequently occurs amongst patients with tuberculosis (TB) and contributes to poor quality of life and treatment outcomes. This study evaluated the construct validity and reliability of the GAD-7 scale in a sample of patients with TB in the Free State Province.

**Methods:**

A pilot study was conducted amongst a convenience sample of 208 adult patients newly diagnosed with drug-susceptible TB attending primary healthcare (PHC) facilities in the Lejweleputswa District in the Free State. A structured interviewer-administered questionnaire comprising social demographic questions and the GAD-7 scale was used. Confirmatory factor analysis was used to investigate the construct validity of the GAD-7 scale. The reliability of the scale was assessed by calculating Cronbach’s alpha.

**Results:**

The analysis showed that a modified two-factor (somatic symptoms and cognitive -emotional symptoms) model, in which the items ‘Not being able to stop or control worrying’ and ‘Worrying too much about different things’ were allowed to covary (Comparative Fit Index: 0.996, Tucker–Lewis Index: 0.993, Root Mean Square Error of Approximation: 0.070, 90% confidence interval: 0.032–0.089), fitted the data better than a unidimensional (generalised anxiety) or an unmodified two-factor model. The indicators all showed significant positive factor loadings, with standardised coefficients ranging from 0.719 to 0.873. The Cronbach’s alpha of the scale was 0.86.

**Conclusion:**

The modified two-factor structure and high internal consistency respectively provide evidence for construct validity and reliability of the GAD-7 scale for assessing GAD amongst patients with TB. Studies are necessary to assess the performance of this brief scale under routine TB programme conditions in the Free State.

## Background

Mental health is increasingly being prioritised globally as well as in South Africa. In 2015, the World Health Organization (WHO) ranked anxiety disorders – with a global prevalence of more than 260 million – as the sixth largest contributor to disability.^1^ In 2009, a nationally representative adult survey in South Africa established that anxiety disorders were the most prevalent 12-month and lifetime disorders.^2^ By 2015, anxiety disorders were associated with 7.2 years lived with disability in South Africa.^1^ Generalised anxiety disorder (GAD) is a type of anxiety disorder characterised by overwhelming anxiety and worry about ordinary situations occuring frequently for at least 6 months.^1,3^ If left untreated, GAD can impair patients’ quality of life and disease treatment outcomes.^4,5,6^ Despite being treatable,^7^ there are concerns that GAD remains largely underdiagnosed and undertreated in primary healthcare (PHC) settings in South Africa.^8^

Tuberculosis (TB) is a communicable disease caused by *Mycobacterium tuberculosis.* It is a major cause of ill health and a leading cause of death from a single infectious agent. In 2019, 10 million people were diagnosed with TB globally, and an estimated 1.2 million and 208 000 deaths were reported amongst human immunodeficiency virus (HIV)-negative and HIV-positive people respectively.^9^ South Africa is ranked amongst the 30 high TB burden countries, accounting for 3.6% (306 000 cases) of the global TB incidence in 2019. In the same year, the country recorded 22 000 deaths amongst HIV-negative people and 36 000 deaths amongst HIV-positive people.

Reviews indicate that anxiety is frequent in patients with TB.^10,11,12^ The prevalence of anxiety in patients with TB ranges between 12% and 70%.^4,13,14^ As was established in an Ethiopian study, the risk for GAD specifically is heightened amongst patients with TB and comorbid HIV.^15^ However, there is a dearth of evidence on the screening and treatment of GAD in patients with TB in South Africa. This could be attributed, in part, to the poor integration of mental healthcare within programmes at the PHC level.^16,17,18^ Consequently, there are hardly any routine screening tools or treatment care models for GAD amongst patients with TB within PHC programmes.^18^

Valid, reliable and easy-to-administer tools are necessary within the TB programme to timeously detect individuals at risk of GAD to facilitate early intervention. The GAD-7 scale is used to identify GAD in individuals and to assess symptom severity.^19,20,21^ Based on the symptom criteria for GAD in the *Diagnostic and Statistical Manual of Mental Disorders Fourth Edition,*^3^ scores range from 0 to 21. Scores between 5 and 9, 10 and 14, and 15 and higher represent mild, moderate and severe anxiety symptoms respectively.^19,20^ The GAD-7 scale has been validated in various populations in developed and developing countries including PHC users in Finland^22^ and Zimbabwe,^23^ college students in Portugal^24^ and Korea^25^ and an adolescent population in Ghana.^26^ In terms of construct validity, the majority of studies have established a unidimensional^27^ factor structure of the GAD-7 scale. However, other research found that a one-factor structure may not always fit the data well,^28^ suggesting a need for a context-specific analysis. Furthermore, studies have reported satisfactory internal consistency of both the English version^27^ and translated versions^23,29,30^ of the GAD-7 with a Cronbach’s alpha value of at least 0.8.^27^ However, there is a dearth of information on the performance of this scale amongst patients with TB. Besides, no study has assessed the performance of the GAD-7 scale in the Free State Province. This study sought to establish the construct validity and reliability of an interviewer-administered GAD-7 scale in a sample of patients with newly diagnosed drug-susceptible TB in the Free State.

## Methods

### Design and setting

A pilot study was conducted amongst patients with TB in the Lejweleputswa District in the Free State Province of South Africa. Eleven PHC facilities were purposefully selected from the district based on a high burden of TB.

### Participant sampling and recruitment

The study population constituted adult patients newly diagnosed with susceptible TB. A convenience sampling strategy was used to select patients aged 18 years and older, who had initiated treatment between 01 May 2019 and 31 October 2019, and were proficient in either English or Sesotho. Patients younger than 18 years, those who were too ill to be interviewed, those on TB re-treatment and those with multidrug-resistant TB were excluded from the study.

Patients were recruited through their attending nurses. The nurses informed them about the study and referred them to trained fieldworkers located in private spaces within the facility premises. Eligible patients provided written informed consent for interviews as well as access to their clinical information.

### Data collection

A structured interviewer-administered questionnaire was used for data gathering. The questionnaire comprised five questions to obtain the patients’ social-demographic and clinical information including sex (male or female), age, marital status (married or unmarried), educational qualification (no formal education, primary, secondary or tertiary) and HIV status (negative, positive or not recorded). The seven-item GAD scale was used to assess GAD in the patients.^19,20^ The patients were asked to indicate how often they experienced anxiety symptoms over two weeks before assessment. These included the following: (1) feeling nervous, anxious or on edge; (2) not being able to stop or control worrying; (3) worrying too much about different things; (4) trouble relaxing; (5) being so restless that it is hard to sit still; (6) becoming easily annoyed or irritable; and (7) feeling afraid as if something awful might happen. Responses were recorded on a 4-point Likert scale as follows: 0 = ‘not at all’; 1 = ‘several days’; 2 = ‘more than half the days’; and 3 = ‘nearly every day’. As with other validation studies in Africa^31,32,33,34^ adapting response sets to improve respondent comprehension, response sets in this study were adjusted such that ‘several days’ was depicted as 1–7 days; ‘half the days’ was depicted as 8–11 days; and ‘nearly every day’ was depicted as 12–14 days.

The research instruments including a consent form and the questionnaire were forward-translated into Sesotho and back-translated to English by two independent translators who discussed discrepancies between original and translated versions with the research team before consensus was reached on the final draft of the translated questionnaire. A team of experienced bilingual fieldworkers conducted face-to-face interviews with the patients in either Sesotho or English. The questionnaire took approximately 10 minutes to complete.

### Analysis

Data from 208 patients with drug-susceptible TB were analysed. The International Business Machines (IBM) Statistical Package for the Social Sciences (SPSS) version 27^35^ was used to analyse the patients’ socio-demographic and clinical characteristics. Discrete variables were presented as frequency counts and percentages, and continuous variables as means and standard deviations (SDs). Construct validity of the GAD-7 scale was investigated by using confirmatory factor analysis (CFA). The CFA models were fitted by using lavaan version 0.5–23^36^ in R version 3.6.0.^37^ The dataset was examined for the CFA requirements of multicollinearity, residual values, multivariate outliers and normality. Only the CFA assumptions of multicollinearity, residual values and multivariate outliers were satisfied. The assumption of normality was violated for several variables probably because the variables were measured on a Likert scale and were thus ordinal rather than continuous in nature. To account for the violation of this assumption, the variables were specified as ‘ordered’ (ordinal variables) when fitting the CFA model, and the diagonally weighted least squares (DWLS) estimator was used. The Comparative Fit Index (CFI) and the Tucker–Lewis Index (TLI) were used to determine whether the model fitted the data better than a more restricted baseline model. The root mean square error of approximation (RMSEA) was used to measure how closely the model represented data patterns. The model’s performance was tested by examining the differences between the expected and actual correlation matrix. Internal consistency of the GAD-7 scale was evaluated by calculating the Cronbach’s alpha.

### Ethical considerations

This study was approved by the Health Sciences Ethics Review Board (UFS-HSD2019/1574/2611) at the University of the Free State. Permission to conduct the study at PHC facilities was provided by the Free State Department of Health. Participation in the research was entirely voluntary. Eligible patients signed consent forms upon being informed about the purpose of the study. All information gathered during the study was handled confidentially, and data were secured in locked cabinets.

## Results

### Sample characteristics

Table 1 shows the sample’s socio-demographic and clinical characteristics. Two-thirds of the sample were male (*n* = 137; 65.9%). Just over half (*n* = 116; 55.8%) of the patients were aged between 18 and 40 years.

**TABLE 1:** Participants’ characteristics (*N* = 208).

Variable	*n*	%
**Sex** Male Female	137 71	65.9 34.1
**Age**†**(years)** 18–30 31–40 41–50 51–60 61 and older	52 64 31 27 34	25.0 30.8 14.9 13.0 16.3
**Marital status** Married Unmarried	68 140	32.7 67.3
**Educational qualification** No formal education Primary school Secondary school Matric or grade 12 Tertiary education	3 53 112 34 6	1.4 25.5 53.9 16.3 2.9
**HIV status** Negative Positive Not recorded	80 118 10	38.5 56.7 4.8
**Symptoms of anxiety** No symptoms Mild symptoms Moderate symptoms Severe symptoms	114 59 25 10	54.8 28.4 12.0 4.8

HIV, human immunodeficiency virus.

† , Mean age (SD): 42.4 (15.2); median age (inter-quartile range): 38.5 (30.3–54.0) years.

Seven in every 10 patients were unmarried (*n* = 140; 67.3%). Slightly more than half had attained secondary school education (*n* = 112; 53.8%). Almost six in every 10 patients were co-infected with HIV (*n* = 118; 56.7%). Based on the criteria for GAD in the *Diagnostic and Statistical Manual of Mental Disorders Fourth Edition*,^3^ just under half of the sample (*n* = 94; 45.2%) had symptoms of GAD. More specifically, 28.4% (*n* = 59) of the patients had mild anxiety symptoms, 12.0% (*n* = 25) had moderate anxiety symptoms and 4.8% (*n* = 10) had severe anxiety symptoms.

### Construct validity of the generalised anxiety disorder-7 scale

Table 2 depicts the Goodness-of-fit indices of models for the GAD-7 scale. The latent factors were standardised, allowing free estimation of all factor loadings. The first model, with only a single latent variable (generalised anxiety) specified, showed adequate CFI (0.966) and TLI (0.949) scores, but a RMSEA value of 0.188 (90% confidence interval [CI]: 0.157–0.220) indicated a poor fit. The second model, with two latent factors specified, that is, somatic symptoms and cognitive-emotional symptoms, showed an improved fit when compared with the first model, with a CFI of 0.981 and a TLI of 0.969. The RMSEA also improved from 0.188 to 0.147 (90% CI: 0.115–0.181), but still indicated a poor model fit. Although the second model was deemed to be an improvement on the first model, the RMSEA value was still not satisfactory. Modification indices suggested that allowing the items ‘Not being able to stop or control worrying’ and ‘Worrying too much about different things’ to covary might lead to an improved model fit. Thus, a third two-factor model was run in which these two variables were allowed to covary. A chi-square difference test showed a statistically significant improvement in model fit between the modified and unmodified two-factor models (χ^2^[1] = 47.192, *p* < 0.001). The fit indices also showed an improved fit, with a CFI of 0.996 and a TLI of 0.993. Furthermore, the RMSEA indicated a good model fit, with a value of 0.070 (90% CI: 0.027, 0.110). As expected, for this final model, the indicators all showed significant positive factor loadings, with standardised coefficients ranging from 0.719 to 0.873 (Table 3). The item means ranged from 0.45 (SD: 0.87) to 1.09 (SD: 1.02) (Table 4). Taken together, the results indicate that a modified two-factor model, with somatic symptoms and cognitive-emotional symptoms as latent factors, that allows the items ‘Not being able to stop or control worrying’ and ‘Worrying too much about different things’ to covary, resulted in the best fit for the data when compared with a single-factor model with only generalised anxiety as an underlying factor, and an unmodified two-factor model. The results also provide evidence for the construct validity of the GAD-7 scale in the sample studied.

**TABLE 2:** Goodness-of-fit indices of models for the generalised anxiety disorder-7 scale (*N* = 208).

Model	*χ* ^2^	DF	CFI	TLI	RMSEA	90% CI
One-factor	116.023*	14	0.966	0.949	0.188	0.157–0.220
Two-factor	71.202*	13	0.981	0.969	0.188	0.115–0.181
Unmodified two-factor	24.01**	12	0.996	0.993	0.070	0.027–0.110

χ^2^, model Chi square; DF, degrees of freedom; CFI, Comparative Fit Index; TLI, Tucker–Lewis Index; RMSEA, root mean square error of approximation; CI, confidence interval.

* , *p* < 0.001;

** , *p* < 0.05.

**TABLE 3:** Unstandardised and standardised factor loadings for the modified two-factor model of the generalised anxiety disorder-7 scale.

Latent factor	Indicator	*B*	SE	*Z*	Beta	Sig
Somatic symptoms	Trouble relaxing	0.840	0.049	17.214	0.840	< 0.001
Somatic symptoms	Being so restless that it is hard to sit still	0.873	0.046	18.905	0.873	< 0.001
Somatic symptoms	Becoming easily annoyed or irritable	0.719	0.054	13.251	0.719	< 0.001
Cognitive-emotional symptoms	Feeling nervous, anxious or on edge	0.734	0.051	14.368	0.734	< 0.001
Cognitive-emotional symptoms	Not being able to stop or control worrying	0.735	0.048	15.440	0.735	< 0.001
Cognitive-emotional symptoms	Worrying too much about different things	0.747	0.047	16.059	0.747	< 0.001
Cognitive-emotional symptoms	Feeling afraid as if something awful might happen	0.819	0.044	18.728	0.819	< 0.001

*B*, unstandardised beta coefficient; SE, standard error; *Z*, standard Z-score; Beta, standardised beta coefficient; Sig, statistical significance.

**TABLE 4:** Descriptive characteristics for observed variables.

Variable	*M*	*SD*	Min	Max
Trouble relaxing	0.51	0.87	0	3
Being so restless that it is hard to sit still	0.45	0.83	0	3
Becoming easily annoyed or irritable	0.82	0.95	0	3
Feeling nervous, anxious or on edge	0.60	0.75	0	3
Not being able to stop or control worrying	0.97	1.01	0	3
Worrying too much about different things	1.09	1.02	0	3
Feeling afraid as if something awful might happen	0.63	0.88	0	3

M, mean; SD, standard deviation; Min, minimum score; Max, maximum score.

### Reliability

The Cronbach’s alpha for the full GAD-7 scale was 0.86, indicating that the scale exhibited acceptable internal consistency in this sample. High correlations were observed between the seven items and the total scores, ranging from 0.57 to 0.70 (Table 5). The sub-scales ‘Somatic symptoms’ and ‘Cognitive-emotional symptoms’ also exhibited satisfactory internal consistency with Cronbach’s alpha of 0.73 and 0.83 respectively.

**TABLE 5:** Generalised anxiety disorder-7 scale item-level values and item–total correlations.

Variable	Correlated item–total correlation	Alpha if item is deleted
Trouble relaxing	0.57	0.84
Being so restless that it is hard to sit still	0.58	0.84
Becoming easily annoyed or irritable	0.57	0.84
Feeling nervous, anxious or on edge	0.58	0.84
Not being able to stop or control worrying	0.70	0.82
Worrying too much about different things	0.70	0.84
Feeling afraid as if something awful might happen	0.64	0.83

Overall Cronbach’s alpha = 0.86.

## Discussion

This study sought to assess the factor structure, constructive validity and reliability of the GAD-7 scale in a sample of patients newly diagnosed with drug-susceptible TB in the Free State Province. As far as can be ascertained, this is the first study to do so. In line with research in England^38^ and the United States of America,^28^ the CFA results established two latent factors underlying the GAD-7 scale. The first latent factor comprised three somatic items including ‘trouble relaxing’, ‘being so restless that it is hard to sit still’ and ‘becoming easily annoyed or irritable’. The second latent factor comprised four cognitive-emotional items including ‘feeling nervous, anxious or on edge’, ‘not being able to stop or control worrying’, ‘worrying too much about different things’ and ‘feeling afraid as if something awful might happen’. Analysis of the scale further established that a modified two-factor model fit the data better compared with a single-factor model or an unmodified two-factor model. These findings thus support the research suggesting that the originally proposed unidimensional factor structure of the GAD-7 scale^19^ may not always provide a good fit to data.

In this study, the GAD-7 scale was translated to Sesotho and was interviewer-administered. Assessment of reliability established that the GAD-7 scale exhibited a Cronbach’s alpha value of 0.86 implying a good internal consistency of the scale. Other studies in African countries involving PHC attendees^23,29^ have also found the GAD-7 scale to have good internal consistency. Accordingly, the GAD-7 scale is a potentially useful tool for the routine screening of GAD within PHC programmes such as TB and could be used to facilitate the timeous identification of patients who might require additional psychosocial evaluation and support during treatment. However, this necessitates appropriate training of health workers and the development of guidelines for the routine screening of patients. Furthermore, given that the scale is brief, simple to score and freely available,^19,20^ future research should explore its suitability for routine administration by non-clinical or lay health workers within TB programmes. Indeed, there is growing evidence that the use of non-specialist health workers is a key strategy for closing the treatment gap within mental healthcare.^39^ Lay health workers would also have to play a key role in the development and validation of culturally relevant screening tools.^23,40^

A strength of this study is that, in the light of the increasing attention being paid to mental health in South Africa, the results highlight the need to assess GAD in patients with TB and can be used to inform future validation research in the Free State and similar settings. However, this study had some limitations. Because of resource and time constraints, the performance of the GAD-7 scale against other tools measuring anxiety disorders could not be assessed in this study setting. Determination of severity of anxiety in this study was based on the symptom criteria for GAD in the *Diagnostic and Statistical Manual of Mental Disorders Fourth Edition.*^3^ More research is needed to confirm the sensitivity and specificity of the GAD-7 scale in this setting, as well as its performance across demographic groups such as sex and age. In addition, there is potential for sampling bias as the patients in this study were conveniently sampled. The results are therefore not generalisable to all patients with TB.

## Conclusion

The CAF results of this pilot study support a modified two-factor structure of the GAD-7 scale. The GAD-7 scale was also found to have construct validity and acceptable internal consistency, implying that it is reliable for use amongst patients with TB. The TB programme in the Free State could explore the feasibility of using the GAD-7 scale as a routine screening tool for GAD. Further validation studies should explore the performance of the GAD-7 compared with other anxiety screening tools under routine or programmatic conditions.
